# Characterising Vocal Function and Laryngeal Structural Alterations in Ehlers–Danlos Syndromes: Insights from a Scoping Review

**DOI:** 10.3390/biology15141099

**Published:** 2026-07-08

**Authors:** Carmen Morales-Luque, Marta González-García, Laura Carrillo-Franco, Adriana Perales-Guerra, Ana Redondo-Fernández, Manuel Víctor López-González, Marc Stefan Dawid-Milner

**Affiliations:** 1 Department of Human Physiology, Faculty of Medicine, University of Málaga, 29010 Málaga, Spain; adrianaperales004@uma.es (A.P.-G.); anaredondofdez@uma.es (A.R.-F.); manuelvictor@uma.es (M.V.L.-G.); msdawid@uma.es (M.S.D.-M.); 2 Biomedical Research Institute of Málaga (IBIMA Plataforma BIONAND), 29010 Málaga, Spain; mgonzalezgarcia@uma.es; 3 Unit of Neurophysiology of the Autonomic Nervous System (CIMES), University of Málaga, 29010 Málaga, Spain; 4 Department of Nursing, Faculty of Health Sciences, University of Málaga, 29071 Málaga, Spain

**Keywords:** Ehlers–Danlos syndrome, dysphonia, vocal fold, cricoarytenoid joint, lamina propria, scoping review

## Abstract

Ehlers–Danlos syndromes (EDSs) are heritable connective tissue disorders caused by defects in collagen and related extracellular matrix proteins. Because the vocal fold (VF) lamina propria is a collagen-dependent structure, the voice might be expected to be affected, yet voice and laryngeal involvement has received comparatively little attention. This scoping review, conducted according to the Joanna Briggs Institute framework and reported following the PRISMA extension for Scoping Reviews (PRISMA-ScR), mapped the available evidence on voice and laryngeal manifestations in adults with EDS. PubMed, Scopus, and Web of Science were searched to 11 March 2026, and thirteen studies were included: four questionnaire-based and nine clinical or instrumental. Self-reported voice complaints were common across EDS and hypermobility spectrum disorder populations, ranging from mild-to-moderate handicap in large cohorts to frequent, fluctuating difficulties in professional singers, although their prevalence varied with population and assessment method. Direct laryngeal examination revealed a recurring substrate of cricoarytenoid joint subluxation or fixation, arytenoid prolapse, and hyolaryngeal instability, together with microvascular and mucosal fragility, characteristically accompanied by preserved VF mobility. Muscle tension dysphonia was the most frequent diagnosis in high-vocal-demand cohorts. Reflux, vocal load, and autonomic comorbidities such as postural orthostatic tachycardia syndrome were frequently reported and may modulate the clinical picture. The evidence indicates that voice and laryngeal involvement is a plausible, multi-level manifestation of EDS that remains under-recognised. The current literature is limited by small, heterogeneous, and largely uncontrolled studies; prospective research using standardised multimodal assessment is needed to clarify its prevalence, mechanisms, and management.

## Simple Summary

Ehlers–Danlos syndromes (EDSs) are inherited conditions in which the body’s collagen—a protein that gives connective tissue its strength—is faulty, leading to overly flexible joints and fragile tissues. Because the vocal folds (VFs) are built largely from collagen, the voice may also be affected, yet this aspect of the condition has received little attention. We reviewed the available studies on voice and laryngeal manifestations in adults with EDS. Voice complaints were common, and examination of the larynx frequently revealed joint instability and tissue fragility, often while the VFs still moved normally. Greater clinical awareness and further research are needed.

## 1. Introduction

The Ehlers–Danlos syndromes (EDSs) are a clinically and genetically heterogeneous group of heritable connective tissue disorders characterised by joint hypermobility, skin hyperextensibility, and tissue fragility. The 2017 international classification recognises thirteen subtypes, defined by defects in the genes encoding collagens and other extracellular matrix proteins [1,2,3]. The hypermobile type (hEDS) is the most common and, uniquely among the subtypes, still lacks a confirmed molecular cause, so that its diagnosis rests on clinical criteria [4,5]. Beyond the musculoskeletal phenotype, EDS is a multisystemic condition associated with chronic pain and fatigue and with comorbidities such as gastrointestinal dysfunction, postural orthostatic tachycardia syndrome (POTS), and mast cell activation syndrome [6,7,8].

The vocal fold (VF) is, in essence, a layered connective tissue structure whose vibratory behaviour depends on collagen. Within the lamina propria, type I and type III collagens are arranged in distinct layers that provide tensile strength and structural support and that, together with elastin, govern the tissue’s biomechanical properties [9,10,11,12,13]. Collagen has been described as essential to the VF’s capacity to withstand the vibratory impact and stretch of phonation [14]. It is therefore biologically plausible that a systemic disorder of collagen should affect the voice.

Despite this rationale, voice and laryngeal involvement is among the least systematically studied aspects of EDS. The existing evidence has accumulated piecemeal, largely as case reports and small series, and dysphonia—characterised by hoarseness, breathy voice quality, vocal fatigue, or voice instability—has even been described as a rare early presenting symptom of the disorder [15]. As EDS itself remains an often-underappreciated condition in which patients frequently consult multiple specialists before receiving a diagnosis [5], voice complaints are at particular risk of being overlooked. To date, no synthesis has specifically mapped the voice and laryngeal manifestations of EDS in adults, and their nature, frequency, and underlying mechanisms remain poorly defined.

This scoping review therefore aims to systematically map the available evidence on voice and laryngeal manifestations in adults with EDS; to describe the assessment methods used and the structural, functional, and self-reported findings reported; and to identify recurring patterns and knowledge gaps that may inform clinical practice and future research.

## 2. Materials and Methods

### 2.1. Methodological Framework

This study was designed as a scoping review in accordance with the Joanna Briggs Institute (JBI) methodological framework. Given the exploratory nature of the research question and the limited, heterogeneous evidence available in this area, a scoping review was considered the most appropriate design. Reporting adhered to the Preferred Reporting Items for Systematic Reviews and Meta-Analyses extension for Scoping Reviews (PRISMA-ScR). To enhance methodological transparency and minimise post hoc decision-making, a predefined review protocol was registered in the Open Science Framework (OSF) on 27 April 2026 and is publicly available at: https://doi.org/10.17605/OSF.IO/EJHXT. At the time of registration, initial database searching and title and abstract screening had already commenced; however, full-text assessment, data charting, and evidence synthesis had not yet been completed.

### 2.2. Eligibility Criteria

Eligibility criteria were established using the Population–Concept–Context (PCC) framework recommended for scoping reviews.

**Population**. Studies involving adults (≥18 years) diagnosed with EDS, regardless of subtype, were deemed eligible. Given the clinical overlap between EDSs and hypermobility spectrum disorders (HSDs), studies including mixed EDS/HSD populations were also considered when relevant voice or laryngeal data were reported. During data charting, studies involving hypermobile EDS (hEDS) were specifically identified because of its clinical prevalence and potential relevance to functional voice complaints. Articles focused exclusively or predominantly on paediatric populations were excluded. Studies including isolated adolescent participants within otherwise adult cohorts were considered eligible.

**Concept**. Eligible studies were required to explicitly evaluate voice-related symptoms, vocal dysfunction, phonatory alterations, or voice and laryngeal manifestations. Accepted approaches comprised clinical examination, instrumental procedures, perceptual assessment, and patient-reported outcome measures. Representative methods included acoustic analysis, laryngoscopy, videostroboscopy, structured questionnaires, or comparable tools. Studies in which voice or laryngeal manifestations were reported only incidentally, without dedicated evaluation, were excluded.

**Context**. Any clinical, outpatient, community, rehabilitation, or research setting was considered eligible, provided that relevant data on voice or laryngeal manifestations were reported.

Only primary empirical articles published in English were included. Review articles, conference abstracts, editorials, commentaries, preprints, and other non-primary sources were excluded, unless they contained original illustrative clinical cases with extractable voice- or laryngeal-related findings. No date restrictions were applied to ensure comprehensive retrieval, particularly in the absence of prior reviews focused on voice and laryngeal manifestations in EDSs.

### 2.3. Search Strategy and Study Selection

A structured literature search was conducted in PubMed, Scopus, and Web of Science, with the final database query completed on 11 March 2026. The strategy was developed based on the pre-specified eligibility criteria and organised according to the PCC model. Terms related to EDSs were combined with keywords addressing voice, phonatory disorders, and laryngeal manifestations through Boolean operators. No context-specific search terms were applied to maximise sensitivity and reduce the risk of missing relevant evidence, as detailed in Table 1. Database syntax was adapted to the technical requirements of each platform where necessary (Supplementary Table S1).

Titles and abstracts were initially screened within each database. Potentially eligible records were subsequently exported to Mendeley Reference Manager (version 2.103.1), where duplicate entries were removed. The remaining references were then organised in Microsoft Excel for full-text eligibility review.

Study selection was performed independently by two reviewers using a structured two-stage process: (1) title and abstract screening, and (2) full-text assessment against the eligibility criteria. Any disagreements were resolved through discussion, with consultation of a third reviewer when required. Reference lists of all included articles were also manually examined to identify relevant publications; however, no further eligible records were retrieved through this process.

### 2.4. Data Extraction and Variables Collected

After final selection, data were charted using a predefined Microsoft Excel form developed for this scoping review. The data collection template was based on the eligibility criteria and refined iteratively to ensure consistency across heterogeneous study designs and reporting formats.

Two reviewers independently completed the initial data charting. The resulting entries were subsequently compared, and any discrepancies were settled by consensus, with adjudication by a third senior reviewer when necessary. No automated tools were used, and no additional information was sought from the original study authors.

To facilitate the synthesis of heterogeneous evidence, the compiled dataset was organised according to the primary mode of voice assessment, with studies classified as questionnaire-based or as involving clinical or instrumental evaluation.

**Variables extracted from all studies**

- *Bibliographic details*: first author and year of publication.
- *Study design*: methodological design reported by the original authors.
- *Study population*: sample size, EDS subtype, and relevant vocal profile when available.
- *Voice manifestations*: reported symptoms, complaints, or perceived vocal disturbances.
- *Associated conditions*: coexisting clinically relevant symptoms or related features accompanying voice complaints.
- *Key findings*: principal outcomes or conclusions concerning voice or laryngeal involvement in EDSs.

**Specific variables for questionnaire-based studies**

- *Voice assessment method*: questionnaire, survey, or other self-reported measure used to collect voice-related information.
- *Speech-language pathologist/pathology (SLP) history*: previous, current, or reported attendance at speech-language or voice therapy.

**Specific variables for clinical or instrumental studies**

- *Clinical assessment method*: examination or procedure used to assess voice-related symptoms or voice and laryngeal manifestations.
- *Laryngeal function*: abnormalities involving VF behaviour, mobility, coordination, or phonatory mechanics.
- *Laryngeal structure*: structural or organic abnormalities affecting laryngeal tissues.
- *SLP intervention*: reported therapeutic intervention or treatment related to voice complaints.

All information was extracted directly from the original publications and restricted to variables explicitly reported by the study authors. No assumptions were made beyond the data provided in the original reports. Given the exploratory purpose of this scoping review, no formal critical appraisal of methodological quality or risk of bias was undertaken. This decision is consistent with JBI methodological guidance for scoping reviews, which does not mandate critical appraisal as a standard component. Accordingly, the findings should be interpreted as a mapping of the available evidence rather than a quality-weighted synthesis, and conclusions should be read considering the methodological heterogeneity of the included studies.

The included studies were synthesised using a structured descriptive approach. Each study was examined individually and summarised according to the predefined data domains. This process enabled identification of recurring patterns, knowledge gaps, and areas of methodological heterogeneity, without quantitative comparison across studies.

## 3. Results

### 3.1. Search Results and Study Selection

A total of 107 records were identified through database searching. Following the application of automated eligibility filters excluding non-human and in vitro studies, 14 records were removed before screening. The remaining 93 records underwent title and abstract screening, leading to the exclusion of 51 records. The remaining references were exported to the Mendeley Reference Manager, where 26 duplicate records were identified and removed, leaving 16 unique reports for full-text eligibility assessment.

**Figure 1:**
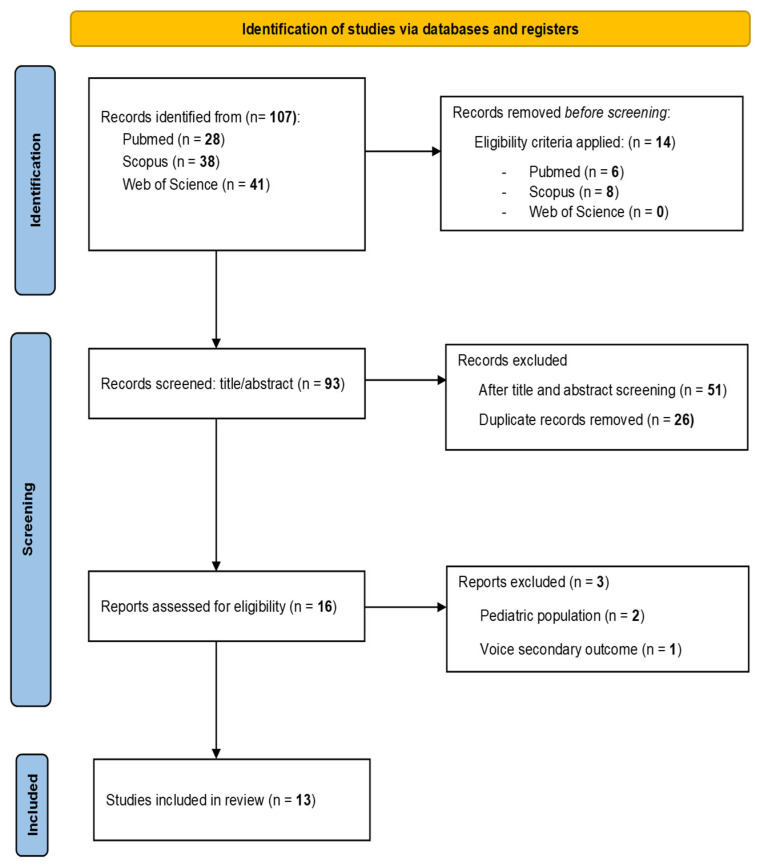
PRISMA 2020 flow diagram of the study selection process.

Three full-text reports were excluded because they involved a paediatric population (*n* = 2) or assessed voice only as a secondary outcome (*n* = 1). Accordingly, 13 studies met the eligibility criteria for inclusion in the final review. The complete study selection process is shown in the PRISMA 2020 flow diagram (Figure 1).

### 3.2. Characteristics of Included Studies

The **13** included studies were published between 2009 and 2025 and were heterogeneous in design, population size, and voice assessment approach. To facilitate synthesis, they were classified into two groups: four questionnaire-based studies examining self-reported voice outcomes (Table 2) and nine clinical or instrumental studies characterising laryngeal structure and function (Table 3). Hypermobile EDS (hEDS) and hypermobility spectrum disorder (HSD) were the most frequently represented diagnoses; vascular (vEDS), classical (cEDS), and periodontal (pEDS) subtypes were additionally documented. Classical-like (clEDS) and dermatosparaxis (dEDS) subtypes were also represented. Results within each subsection are presented in chronological order and restricted to outcomes explicitly reported in the original publications.

#### 3.2.1. Self-Reported Voice-Related Findings

Across four questionnaire-based studies—three cross-sectional surveys and one mixed-method study, with sample sizes ranging from 71 to 1620 participants—self-reported voice complaints were consistently identified in adults with EDS/HSD, although prevalence and severity varied according to study population and assessment methods. **Williams et al.** [16] found a mean voice handicap index (VHI) score of 31.99 (standard deviation (SD) 24.36), reflecting an overall mild-to-moderate level of voice-related impairment, with scores distributed across severity levels: 53.4% mild, 32.6% moderate, and 14.6% severe. No significant differences in total VHI scores were found across diagnostic subgroups; however, participants with hEDS reported poorer perceived voice quality, described as “creaky and dry.”

Higher rates of voice complaints were apparent in symptomatic or high-vocal-demand populations. **Jeffery et al.** [17] reported that 74.6% of professionally trained singers with HSD/hEDS experienced vocal fatigue, instability, transient voice loss, reduced projection, and pain during phonation, frequently interfering with singing performance and daily communication. Similarly, **Venkatraman et al.** [18] documented lifetime voice complaints in 86.9% of adults with EDS and current dysphonia in 91.5% over a period of 12 months; hoarseness, raspy or strained voice quality, and frequent throat clearing were among the most reported manifestations, together with substantial social, occupational, and academic limitations. In contrast, in a clinic-based cohort that included an internal control group, **Menton et al.** [19] observed comparable rates of communication-interfering hoarseness between adults with hEDS/HSD (22.5%) and controls (20.3%), with no statistically significant differences between groups; among symptomatic individuals, 52.3% reported hoarseness on more than two days per month.

Despite the high prevalence of self-reported voice complaints, healthcare utilisation remained limited. **Jeffery et al.** [17] noted previous SLP attendance in only 26% of symptomatic singers and laryngology assessment in 13%. **Venkatraman et al.** [18] similarly found that only 20.3% of participants with current voice complaints had sought SLP services.

Beyond these complaints, several studies reported concurrent laryngeal and systemic conditions. **Williams et al.** [16] identified dysphagia (79.4%), laryngopharyngeal reflux (LPR) (86.1%), and temporomandibular joint dysfunction, while **Menton et al.** [19] recorded swallowing-related symptoms in 33.9% of participants and inspiratory noise episodes suggestive of laryngeal dysfunction symptoms in 27%. **Jeffery et al.** [17] documented associations with temporomandibular joint (TMJ) dysfunction, gastro-oesophageal reflux disease (GERD), and dysautonomia or POTS in singers with HSD/hEDS. **Venkatraman et al.** [18] additionally identified higher frequencies of immune-related disorders (35.7% vs. 19.2%) and oesophageal reflux (45% vs. 24.5%) among participants with voice complaints.

**Table 2:** Overview of self-reported voice-related findings in adults with EDS/HSD.

No.	Author (Year)	Study Design	Study Population	Voice Assessment Method	Voice Manifestations	Associated Conditions	SLP History	Key Findings
**1**	**Williams et al. (2023)** **[16]**	Cross-sectional survey study	Adults with hEDS/cEDS/HSD (*n* = 1620)	VHI-30 (Online PROMs)	Dysphonia	Dysphagia; reflux/LPR; TMJ disorders	Not reported	Mild–moderate voice impact with no differences across diagnostic groups
**2**	**Jeffery et al. (2024)** **[17]**	Mixed-method study	Adults with HSD/hEDS and professional singing training (*n* = 71; from 156 singers in a survey cohort of 276 participants)	Online survey (closed and open-ended questions) + Thematic analysis	Voice difficulties (fatigue, instability, voice loss, pain)	TMJ; GERD/reflux; Dysautonomia/POTS	Limited reported use of speech therapy	Vocal unreliability and functional impact in singers with HSD/hEDS
**3**	**Menton et al. (2024)** **[19]**	Cross-sectional survey study	Adults with hEDS/HSD (*n* = 289) + controls (*n* = 74)	Clinic-based questionnaire (REDCap) + VHI-10 (symptom-triggered)	Dysphonia (hoarseness)	Dysphagia; Laryngeal dysfunction symptoms	Not reported	Dysphonia prevalence did not differ between hEDS/HSD and controls
**4**	**Venkatraman et al. (2025)** **[18]**	Cross-sectional survey study	Adults with hEDS, cEDS, clEDS, vEDS, and unspecified EDS (*n* = 437; voice-analysis subgroup from a cohort of 478 participants)	Online voice questionnaire for EDS population	Hoarseness; Raspy and strained voice	Immune disorders; Esophageal reflux	Limited reported use of speech therapy	Frequent voice problems and social/work limitations in adults with EDS

***Note.* EDS** = Ehlers–Danlos syndrome; **HSD** = hypermobility spectrum disorder; **SLP** = speech-language pathologist; **hEDS** = hypermobile Ehlers–Danlos syndrome; **cEDS** = classical Ehlers–Danlos syndrome; **VHI-30** = Voice Handicap Index-30 (30-item version); **PROMs** = patient-reported outcome measures; **LPR** = laryngopharyngeal reflux; TMJ = temporomandibular joint; **GERD** = gastro-esophageal reflux disease; **POTS** = postural orthostatic tachycardia syndrome; **REDCap** = Research Electronic Data Capture; **VHI-10** = Voice Handicap Index-10 (10-item version); **clEDS** = classical-like Ehlers–Danlos syndrome; **vEDS** = vascular Ehlers–Danlos syndrome.

#### 3.2.2. Clinical and Instrumental Voice-Related Findings

Nine clinical and instrumental studies—three case reports, four retrospective case series, and two case series, with sample sizes ranging from 1 to 21 participants—documented a spectrum of structural and functional laryngeal abnormalities in adults with EDS. Hoarseness or dysphonia constituted the predominant vocal presentation across the majority of included studies, with additional manifestations comprising aphonia, vocal fatigue, breathiness, roughness, strained voice quality, and reduced vocal range. In the studies by **Sharma et al.** [20] and **Yan et al.** [21], muscle tension dysphonia (MTD) was the most frequently assigned clinical diagnosis among participants from high-vocal-demand populations, while **Lam et al.** [22] reported voice-related symptoms (encompassing dysphonia and mutism) in 42.9% of a specialist laryngology cohort of 21 adults.

At the structural and functional level, findings ranged from microvascular and tissue-level abnormalities to articular instability and phonatory dysfunction. **Desuter et al.** [23] identified right VF epithelial rupture, Reinke’s space haemorrhage, and laryngopharyngeal microaneurysms on narrow-band imaging in one adult with hEDS, with preserved VF mobility and full clinical and laryngoscopic resolution. **Du and Tan** [24] described a haemorrhagic true VF polyp and a presumed microaneurysm in a patient with vEDS, with early haemorrhagic recurrence after initial microsurgical excision. **George et al.** [25] reported two distinct laryngeal presentations in adults with pEDS: bilateral vocal cord sulci in one patient, and a cricoarytenoid joint (CAJ) abnormality (with magnetic resonance imaging (MRI)-confirmed subglottic stenosis) in the other. **Arulanandam et al.** [26] reported heterogeneous laryngeal manifestations in nine adults with hEDS and cEDS—VF scissoring, bilateral arytenoid prolapses, and CAJ subluxation or fixation—with unilateral or bilateral VF immobility in the most severely affected cases. **Birchall et al.** [27] found paradoxical VF motion and CAJ subluxation (the latter initially presenting as apparent VF paralysis) in two adults with hEDS. **Lam et al.** [22] documented arytenoid prolapses or subluxation and hyoid subluxation in 28.6% of 21 adults, framing these within the concept of hyolaryngeal skeletal complex instability.

More recent studies further characterised phonatory and structural abnormalities through videostroboscopy and dynamic imaging. **Sharma et al.** [20] observed glottic closure abnormalities on videostroboscopy—including posterior glottic chink and hourglass configuration—alongside bilateral midfold swellings and VF oedema, predominantly in professional voice users. **Yan et al.** [21] similarly detected impaired glottal closure, abnormal VF adduction, and asynchronous mucosal wave on stroboscopic evaluation, with vocal nodules and VF oedema as concomitant structural findings. **Menton et al.** [28] confirmed phonation-dependent left arytenoid subluxation through dynamic computed tomography (CT) imaging in one adult with hEDS presenting with VF hypomobility and glottic insufficiency. Despite this range of structural and articular abnormalities, VF mobility was preserved in most cases.

SLP intervention was described in five studies. **Birchall et al.** [27] reported vocal tract rebalancing, vocal efficiency strategies, and head and neck relaxation techniques, with hyaluronic acid VF augmentation additionally performed in one case. **Sharma et al.** [20] employed voice therapy as the primary approach for MTD, with steroid injections in one patient with persistent VF oedema following prior microsurgical excision. **Yan et al.** [21] implemented individualised programmes of two to ten sessions incorporating resonant voice techniques, vocal care, breathing retraining, and inspiratory muscle strength training. **Menton et al.** [28] combined hyaluronic acid augmentation with structured voice therapy; after six sessions, breathiness and asthenia improved while moderate roughness and strain persisted upon perceptual evaluation. **Arulanandam et al.** [26] referred selected patients for voice and speech therapy without further specification.

**Table 3:** Overview of clinical and instrumental voice-related findings in adults with EDS.

No.	Author (Year)	Study Design	Study Population	Clinical Assessment Method	Laryngeal Function	Laryngeal Structure	Voice Manifestations	Associated Conditions	SLP Intervention	Key Findings
**1**	**Desuter et al. (2009)** **[23]**	Case report	Adult with hEDS (*n* = 1)	Indirect laryngoscopy; NBI; VF biopsy + electron microscopy	Preserved VF mobility	VF epithelial rupture; Reinke’s space haemorrhage; Laryngopharyngeal microaneurysms	Sudden aphonia	TMJ dislocation	Not reported	Acute aphonia with VF injury and microvascular aneurysms in hEDS
**2**	**Du & Tan** **(2013)** **[24]**	Case report	Adult with vEDS (*n* = 1)	Rigid laryngoscopy; Microscopic direct laryngoscopy	Preserved VF mobility	Hemorrhagic VF polyp; VF microaneurysm (presumed)	Hoarseness; Raspy voice; Reduced vocal range	Globus sensation	Not reported	Persistent hoarseness and recurrent haemorrhagic VF lesions in vEDS
**3**	**George et al. (2016)** **[25]**	Case series	Adults with pEDS (*n* = 2)	Clinical examination; MRI	Not reported	Bilateral vocal cord sulci; Cricoarytenoid joint abnormality; Subglottic stenosis (MRI)	Hoarseness	Not reported	Not reported	Unexpected chronic hoarseness with laryngeal involvement in pEDS
**4**	**Arulanandam et al. (2017)** **[26]**	Retrospective case series	Adults with hEDS/cEDS (*n* = 9)	FNE; Microlaryngoscopy; Laryngeal EMG; GRBAS	VF scissoring; Unilateral/Bilateral VF immobility	Bilateral arytenoids prolapse; Cricoarytenoid joint subluxation/fixation	Dysphonia (hoarseness, vocal fatigue)	Dysphagia; Throat pain; Reflux/LPR; Dysautonomia	Voice/Speech therapy referral	Subtle laryngeal abnormalities underlying dysphonia in EDS
**5**	**Birchall et al. (2021)** **[27]**	Illustrative clinical cases	Adults with hEDS (*n* = 2)	Stroboscopy	Paradoxical VF motion; VF dysfunction mimicking paralysis	Cricoarytenoid joint subluxation	Hoarseness	Reflux-related laryngeal inflammation; Upper airway dysfunction	Targeted speech therapy (vocal tract rebalancing)	Functional laryngeal instability presenting as severe hoarseness in hEDS
**6**	**Lam et al.** **(2022)** **[22]**	Retrospective clinical cases series	Adults with hEDS/dEDS (*n* = 21)	Retrospective laryngology clinic review + PROMs	Not reported	Hyoid subluxation; Arytenoids prolapse/subluxation	Dysphonia; Mutism	Dysphagia; Globus; Choking sensation	Not reported	Hyolaryngeal dysfunction emerging as a recurrent feature in hEDS
**7**	**Sharma et al. (2024)** **[20]**	Retrospective case series	Adults with hEDS (*n* = 9; 6 singers)	Videostroboscopy	Preserved VF mobility; Symmetric mucosal wave; Posterior chink/hourglass closure	Bilateral midfold swellings; VF oedema	Dysphonia (MTD; hoarseness; vocal strain)	Dysphagia; GERD/LPR; POTS	Voice therapy	Glottic closure abnormalities and VF lesions in dysphonic hEDS singers
**8**	**Yan et al.** **(2025)** **[21]**	Retrospective case series	Adults with EDS and high vocal demands (*n* = 4)	Stroboscopy	Impaired glottal closure; Abnormal VF adduction; Asynchronous wave	Vocal nodules; VF oedema	Dysphonia (vocal hyperfunction, reduced vocal range)	LPR/GERD; POTS; Dyspnea	Individualised voice therapy	Hyperfunctional voice patterns in high voice users with EDS
**9**	**Menton et al. (2025)** **[28]**	Case report	Adult with hEDS (n = 1)	Stroboscopy; Dynamic CT	Left VF hypomobility; glottic insufficiency	Left arytenoid subluxation	Hoarseness; Breathiness; Roughness	Dysphagia; dyspnea/dizziness while talking	Structured voice therapy with SLP follow-up	Dynamic arytenoid subluxation during phonation in hEDS

***Note***. **SLP** = speech-language pathologist; **hEDS** = hypermobile Ehlers–Danlos syndrome; **NBI** = narrow-band imaging; **VF** = vocal fold; **TMJ** = temporomandibular joint; **vEDS** = vascular Ehlers–Danlos syndrome; **pEDS** = periodontal Ehlers–Danlos syndrome; **MRI** = magnetic resonance imaging; **cEDS** = classical Ehlers–Danlos syndrome; **FNE** = flexible naso-endoscopy; **EMG** = electromyography; **GRBAS** = Grade, Roughness, Breathiness, Asthenia, Strain scale; **LPR** = laryngopharyngeal reflux; **EDS** = Ehlers–Danlos syndrome; **dEDS** = dermatosparaxis Ehlers–Danlos syndrome; **PROMs** = patient-reported outcome measures; **MTD** = Muscle Tension Dysphonia; **GERD** = gastroesophageal reflux disease; **POTS** = postural orthostatic tachycardia syndrome; **CT** = computed tomography.

Associated conditions were reported in seven of the nine studies. LPR or GERD and dysphagia-related complaints were the most recurrently documented comorbidities, each identified in four studies (Reflux: **Arulanandam et al.** [26], **Birchall et al.** [27], **Sharma et al.** [20], **Yan et al.** [21]; Dysphagia: **Arulanandam et al.** [26], **Lam et al.** [22], **Sharma et al.** [20], **Menton et al.** [28]). Dysautonomia or POTS were recorded in three studies (**Arulanandam et al.** [26], **Sharma et al.** [20], **Yan et al.** [21]). Respiratory and upper airway manifestations were noted in two studies: inspiratory airway compromise and oesophageal dysmotility in one (**Birchall et al.** [27]), and dyspnea, respiratory muscle weakness, and inducible laryngeal obstruction in **Yan et al.** [21]. **Arulanandam et al.** [26] also reported a broader systemic profile—chronic cystitis, bowel hypersensitivity, abdominal symptoms, chronic throat pain, and airway scarring or stenosis—in selected cases. **Desuter et al.** [23] noted recurrent temporomandibular joint dislocation as the initial presenting feature prompting EDS evaluation; **Menton et al.** [28] recorded dyspnea and dizziness during phonation alongside pre-diagnostic generalised joint hypermobility.

## 4. Discussion

The studies included in this scoping review provide accumulating evidence suggesting that voice and laryngeal manifestations may represent a clinically relevant feature of EDSs. Over more than fifteen years of research, these alterations have been documented in diverse populations, using a variety of assessment methods and involving multiple EDS subtypes. Self-reported studies indicate that voice complaints are common and often associated with meaningful functional limitations, while clinical and instrumental investigations show that direct laryngeal examination can reveal structural and functional abnormalities that are not always captured by patient-reported outcomes alone.

Taken together, the available evidence suggests that voice and laryngeal involvement is a plausible and clinically relevant manifestation of EDS rather than an incidental finding, consistent with the underlying connective tissue fragility that characterises the disorder. The available data indicate that this involvement may affect the laryngeal apparatus at multiple levels—histological, articular, and vascular—although the precise mechanisms and their relative contributions remain to be established. Additional factors, such as LPR, autonomic dysfunction, and high vocal demand, may further modulate the clinical expression of these manifestations. Despite the accumulating evidence, voice and laryngeal involvement remains largely overlooked in routine clinical care and continues to receive limited attention within the scientific literature.

### 4.1. Vocal Burden in EDS: Insights from Questionnaire-Based Studies

Voice remains one of the least systematically investigated dimensions of the multisystemic phenotype of EDS, whose molecular and clinical basis is otherwise comparatively well characterised [2]. The questionnaire-based studies included in this review are the first to quantify this burden in large cohorts, and their consistent findings indicate that voice complaints are both common and clinically relevant in adults with EDS. **Study 1 (Table 2)** [16], which included the largest cohort in this block and employed validated assessment instruments, documented voice-related impairment across a range of severity levels. The predominance of the physical domain of the VHI over its functional and emotional domains suggests that dysphonia in EDS is primarily experienced as a sensorimotor disturbance affecting vocal production. **Study 2 (Table 2)** [17] offers a complementary perspective by focusing on professionally trained singers with HSD/hEDS, a population in which subtle vocal dysfunction is likely to be recognised at an earlier stage. In this group, the most distinctive feature was not progressive deterioration but symptom unpredictability, characterised by transient voice loss, phonatory instability, and vocal fatigue. Such a fluctuating presentation may contribute to under-recognition in clinical practice. Nevertheless, professional voice use represents an important confounding factor. Sustained vocal loading can independently increase self-reported vocal effort and discomfort [29], and because the study relied exclusively on self-reported data, it is not possible to determine whether the reported symptoms were attributable to EDS, professional vocal demand, or an interaction between both factors. **Study 4 (Table 2)** [18] further suggests that vocal dysfunction may extend beyond subjective experience to affect daily functioning. Collectively, these three studies point to the conclusion that vocal burden in EDS is both real and clinically meaningful across different populations and real-world contexts.

**Study 3 (Table 2)** [19] appears to diverge from this pattern, reporting no significant differences in voice-related measures between patients with hEDS/HSD and controls. However, this finding is more plausibly explained by methodological factors than by the absence of a true association. The comparator group consisted of symptomatic individuals attending the same clinic who did not meet diagnostic criteria for hEDS/HSD, thereby reducing the study’s ability to detect meaningful between-group differences. Furthermore, voice assessment was restricted to a single binary question regarding hoarseness, while the Voice Handicap Index-10 (VHI-10) was administered only to participants who had already reported symptoms. The performance characteristics of the VHI-10 should also be considered when interpreting these findings. Its sensitivity reaches only 29% against acoustic analysis (and 48% against perceptual-auditory assessment) in population-based settings [30], and its correlation with objective acoustic measures is only moderate [31].

These observations highlight that self-reported and instrumental assessments capture different dimensions of vocal function. More broadly, this limitation applies to all studies included in this block. Although questionnaire-based investigations provide valuable information regarding perceived burden and functional impact, the absence of laryngoscopic, perceptual, or acoustic assessment prevents a more detailed characterisation of the nature and potential mechanisms underlying vocal dysfunction in individuals with EDS.

### 4.2. Structural and Functional Laryngeal Alterations in EDSs

Self-reported measures capture the perceived impact of vocal dysfunction but cannot fully characterise its underlying origin. Whereas the questionnaire-based studies document the subjective burden of vocal dysfunction, direct examination of the larynx reveals the structural and functional substrate that may underlie this symptomatology. The nine studies included in this block are heterogeneous in design and sample size, yet their findings converge on a coherent clinical picture that extends the characterisation of voice and laryngeal involvement in EDSs beyond the mere self-reporting of symptoms.

Articular instability of the laryngeal skeleton is the most consistently documented finding. The CAJ is a small multiaxial synovial joint whose rotating, rocking, and gliding movements position the arytenoid cartilages and thereby govern VF abduction and adduction [32]; its functional integrity therefore depends on competent periarticular connective tissue support. Nevertheless, a conceptually important distinction exists between the alterations described. Some authors report subluxation or fixation of the CAJ, defined as a partial loss of contact between the articular surfaces, with complete loss corresponding to frank dislocation [33]—an abnormality documented by endoscopic examination in **Studies 4 and 5 (Table 3)** [26,27] and, in **Study 9 (Table 3)** [28], confirmed during phonation by dynamic CT. More recently, laryngeal ultrasonography has been shown to depict the CAJ and its rotating, rocking, and gliding movements in real time, albeit with the joint cavity delineated in only a minority of cases [34]. **Study 6 (Table 3)** [22] describes positional prolapse of the arytenoids, a phenomenon in which insufficient periarticular support allows these structures to behave in a hyperlax manner during dynamic tasks, without necessarily implying intrinsic joint failure [35]. Both entities probably share a common origin in connective tissue fragility but may represent different manifestations of the failure of laryngeal support mechanisms. **Study 4 (Table 3)** [26] described both arytenoid prolapse and cricoarytenoid subluxation/fixation in different patients within the same clinical series, suggesting that these alterations should be understood as complementary manifestations of voice and laryngeal involvement in EDS rather than as mutually exclusive entities. In this context, the concept of hyolaryngeal skeletal complex instability introduced in **Study 6 (Table 3)** [22], which integrates hyoid and arytenoid subluxations as concurrent expressions of a systemic connective tissue vulnerability, offers a particularly useful integrative perspective, shifting the clinical focus from an isolated joint pathology towards consideration of the hyolaryngeal complex as a functional unit.

The CAJ has, moreover, long been recognised as the laryngeal site most frequently involved in connective tissue disorders more broadly, although the available evidence derives predominantly from inflammatory arthropathies such as rheumatoid arthritis rather than from heritable disorders of collagen [36]. Several converging lines of evidence suggest that, in EDSs, articular displacement reflects a constitutionally vulnerable joint rather than the magnitude of any external force. In ex vivo human larynges, even high-force simulated intubation approximating the maximum force attainable under extreme conditions failed to dislocate the CAJ or to disrupt its capsule and ligament [37], and arytenoid dislocation following endotracheal intubation remains rare in the general surgical population, with a pooled incidence of approximately 0.09% [38]. Against this background, the occurrence of arytenoid subluxation or dislocation in EDS—at times spontaneously or after seemingly trivial mechanical stress—is more plausibly attributable to intrinsic joint instability. Consistent with this, spontaneous arytenoid dislocation has been linked to congenital or acquired arytenoid instability that facilitates displacement after even minor trauma [39], a mechanism that aligns closely with the connective tissue fragility characteristic of EDS.

A clinically relevant observation that recurs across this group of studies is the preservation of VF mobility despite the presence of demonstrable structural and articular alterations. This dissociation suggests that the deterioration of laryngeal function in EDSs does not appear to depend primarily on a primary alteration of the neural mechanisms of adduction and abduction, but rather on biomechanical changes associated with the loss of structural stability. The exception described in **Study 9 (Table 3)** [28], consisting of unilateral hypomobility associated with a confirmed arytenoid subluxation, illustrates how functional consequences may emerge when the articular alteration reaches a sufficient degree of mechanical compromise.

Relatedly, the available evidence indicates that structural laryngeal alterations do not translate uniformly into clinically significant vocal dysfunction. Although numerous studies describe patients with objective anatomical findings accompanied by dysphonia, **Study 6 (Table 3)** [22] showed that vocal dysfunction was not present in all individuals with documented hyolaryngeal instability. In their cohort, the hyoid and arytenoid alterations formed part of a broader pattern of hyolaryngeal dysfunction, whereas voice-related symptoms affected fewer than half of the patients. This finding suggests that anatomical instability, on its own, may be insufficient to determine the functional vocal outcome. Additional factors—such as vocal demand, the severity of tissue involvement, compensatory mechanisms, or the coexistence of other systemic alterations—could modulate the clinical expression of voice and laryngeal involvement. This variability may explain why some individuals present with marked dysphonia and relatively subtle anatomical findings, whereas others show evident structural alterations with limited vocal repercussion.

Other studies document microvascular and mucosal fragility as a distinct but complementary expression of connective tissue vulnerability. These findings (Studies 1 and 2, Table 3 [23,24]) suggest that the laryngeal mucosa partakes of the systemic fragility characteristic of EDSs. From a clinical standpoint, these lesions may have persistent functional consequences beyond their apparent resolution, including lasting alterations of the mucosal wave [40]—a consideration of relevance in a disorder in which vascular vulnerability forms part of the pathological mechanism itself.

Finally, the videostroboscopic findings described in **Studies 7 and 8** [20,21]—including glottic closure abnormalities, mucosal wave irregularities, and bilateral phonotraumatic lesions—introduce a functional dimension that warrants careful interpretation. MTD, the most frequent diagnosis in these studies, is traditionally defined as a primary functional disorder in the absence of underlying organic pathology [41]. However, in patients with EDS, in whom objective examination reveals articular instability, mucosal fragility, and structural alterations of the VFs, this classification requires critical appraisal. The available findings raise the possibility that, at least in some patients, the hyperfunctional patterns observed represent compensatory responses to inefficient laryngeal biomechanics or to insufficient structural stability, rather than an exclusively functional disorder. Although the current evidence does not allow causal relationships to be established, this hypothesis has potentially relevant implications for the diagnostic interpretation and therapeutic management of dysphonia in this population.

### 4.3. From Collagen to the Mucosal Wave: A Histological Substrate for Dysphonia in EDS

The phonatory function of the VF rests on the layered architecture of its lamina propria, in which collagen is a principal structural determinant. Histological studies of the human VF have shown that type I and type III collagens are distributed across the lamina propria in complementary patterns [10,11,42]: type III is thought to maintain the structural framework while type I provides tensile strength [11], with their relative proportions varying across the superficial, intermediate, and deep layers [13,43]. This collagenous scaffold underpins the biomechanical behaviour of the VF during phonation; collagen, together with elastin, governs the tensile elastic properties of the VF cover and ligament [12] and acts as the principal load-bearing reinforcement of the extracellular matrix [44]. Because the mucosal wave depends on the pliability and tensile integrity of these superficial layers, alterations in the amount or organisation of collagen would be expected to degrade vibratory function [9,14].

Because EDS arises from defects in the biosynthesis, organisation, or supramolecular assembly of collagen—including type I and type III collagens that predominate in the VF lamina propria [2,3]—it is biologically coherent that this group of disorders should compromise the very tissue whose vibratory function depends on collagen integrity. This interpretation is explicitly supported by several included studies **(Studies 3, 4, 5 and 6, Table 3** [22,25,26,27]) which attributed dysphonia in EDSs to collagen alterations affecting both the lamina propria and the periarticular structures of the larynx.

Taken together, these observations support a unifying mechanistic framework in which the connective tissue defect of EDSs undermines vocal function through two complementary routes: degradation of the lamina propria collagen that sustains the mucosal wave and weakening of the periarticular and capsular collagen that stabilises the CAJ (Section 4.2). This dual substrate—one mucosal and vibratory, the other articular and biomechanical—may account for the heterogeneous phenotype observed across the included studies, in which dysphonia can accompany either subtle mucosal change or overt arytenoid displacement, and helps to explain why structural findings and vocal dysfunction do not map onto one another in a simple fashion. It should be emphasised, however, that this dual-substrate framework constitutes a biologically plausible interpretive construct informed by the connective tissue pathophysiology of EDSs; a direct mechanistic link between collagen defects, mucosal wave degradation, and articular instability has not been formally demonstrated by the studies included in this review and should be regarded as a hypothesis requiring prospective validation.

### 4.4. Systemic Comorbidities and the Autonomic Context

Beyond the laryngeal apparatus itself, the included studies repeatedly situated voice complaints within a broader systemic context. Dysautonomia and POTS were reported as associated conditions in **Studies 4, 7, and 8 (Table 3)** [20,21,26] and **Study 2 (Table 2)** [17]. This is consistent with the wider literature, in which POTS and mast cell activation syndrome are recognised systemic comorbidities of EDSs and hypermobility spectrum disorders [7,8].

One included study went further in proposing a direct laryngeal link: **Study 7 (Table 3)** [20] suggested that, given the association of EDSs with disorders of autonomic regulation including POTS, vagal nerve sensitivity might act as a potential cause of laryngospasm in this population.

Outside the EDS literature, a separate body of work has examined the relationship between the autonomic nervous system (ANS) and the larynx in functional and hyperfunctional voice disorders. In non-EDS populations—including patients with vocal hyperfunction and professional singers without voice complaints—measures of autonomic function such as skin conductance and heart rate variability have been found to correlate with vocal function and with the tone of the laryngeal and vocal tract musculature [45,46,47]. Whether such an autonomic–laryngeal relationship contributes to the MTD and hyperfunctional patterns observed in EDS (Section 4.2) has not, to our knowledge, been investigated directly; it remains an open question that is particularly pertinent given the established overlap between EDS and dysautonomia.

### 4.5. Reflux and Vocal Load as Modulating Factors

Two further factors may modulate the clinical expression of voice and laryngeal involvement in EDS and complicate its interpretation. The first is laryngopharyngeal and gastro-oesophageal reflux, which was among the most frequently reported associated conditions across **Studies 4, 5, 7, and 8 (Table 3)** [20,21,26,27] and **Study 1 (Table 2)** [16]. Reflux is not specific to EDS and can independently affect the voice: a systematic review of human VF tissue exposed to reflux content concluded that laryngopharyngeal reflux induces histological and functional alterations of the VF tissue, including inflammatory infiltration and proteolysis of cell-junction proteins [48], and in patients with suspected reflux, dysphonia and vocal tract discomfort symptoms correlate with one another and improve following proton pump inhibitor therapy [49]. Reflux therefore represents a potential confounder when attributing dysphonia in EDSs to the connective tissue defect itself.

The second factor is vocal load. Several included studies—**Studies 7 and 8 (Table 3)** [20,21] and **Study 2 (Table 2)** [17]—were specifically drawn from professional voice users and singers, and their authors explicitly acknowledged the difficulty of distinguishing the effect of high vocal demand from that of EDS itself. Consistent with this concern, sustained vocal loading has been shown experimentally to increase self-reported vocal effort and discomfort ([29]; Section 4.1). Because much of the available evidence rests on self-report, the relative contributions of EDS, professional vocal demand, and their interaction often cannot be disentangled.

### 4.6. Limitations of the Evidence Base and Future Directions

Several limitations of the current evidence base temper these conclusions. With respect to the search strategy, the review was restricted to PubMed, Scopus, and Web of Science; although these databases were selected for their broad and complementary coverage of the peer-reviewed biomedical literature, representing the primary sources for health sciences research, the exclusion of databases such as EMBASE or CINAHL means that some relevant studies may not have been captured. The available literature is small and methodologically heterogeneous: the clinical and instrumental studies consist of case reports and small retrospective series (one to twenty-one participants), whereas the questionnaire-based studies, although larger, rely on self-report and on samples recruited largely through patient organisations, which may favour the participation of symptomatic individuals—a concern that applies particularly to **Studies 1 and 4 (Table 2)** [16,18]. Furthermore, the predominance of case reports and small clinical series raises the possibility of publication bias, as cases with striking or unusual findings may be more likely to be reported than those with subtle or absent laryngeal involvement. Selection bias is an additional concern: samples recruited through patient organisations or specialist laryngology clinics are likely to over-represent individuals with more severe or complex presentations, potentially overstating the prevalence and severity of voice and laryngeal involvement in the broader EDS population. Adequate control groups were generally lacking; **Study 3 (Table 2)** [19], the single study that included an internal comparison group, used symptomatic clinic attendees who did not meet diagnostic criteria for hEDS/HSD, which limited its ability to detect between-group differences. The marked heterogeneity of assessment methods—ranging from patient-reported outcome measures to laryngoscopy, videostroboscopy, and dynamic CT—precluded any quantitative synthesis, and few studies combined self-report with instrumental, perceptual, and acoustic evaluation, so that the relationship between structural findings and vocal function remains largely uncharacterised.

These gaps define clear priorities for future research. Prospective studies in molecularly or clinically confirmed EDS cohorts, stratified by subtype and employing standardised multimodal assessment that combines perceptual, acoustic, laryngoscopic and patient-reported measures, are needed to establish the prevalence of voice and laryngeal involvement and to clarify the relationship between structure and function. Multicentre longitudinal designs would be particularly valuable to overcome the limitations of small single-centre series and to enable subtype-specific analyses with adequate statistical power. The development of objective voice biomarkers and the incorporation of standardised videostroboscopic protocols and high-speed laryngeal imaging into future protocols would further enhance the characterisation of vibratory function in this population. The contribution of potential modifiers—reflux, vocal load, and autonomic dysfunction—warrants dedicated investigation, as does the reinterpretation of MTD in this population (Section 4.2, Section 4.4 and Section 4.5). Emerging imaging modalities, including dynamic CT—as described in **Study 9 (Table 3)** [28]—and laryngeal ultrasonography of the CAJ [34], may help to characterise articular involvement in vivo. Finally, supportive evidence lies beyond the boundaries of the present review: for example, **Angwin et al.** [50] reported vocal dysfunction in 38% of a molecularly confirmed periodontal EDS cohort, corroborating the periodontal EDS findings of **Study 3 (Table 3)** [25], although this report did not meet the inclusion criterion of a dedicated voice or laryngeal evaluation.

## 5. Conclusions

This scoping review maps a small but converging body of evidence suggesting that voice and laryngeal involvement may represent a clinically relevant manifestation of EDS rather than an incidental finding, although the available evidence does not yet permit definitive conclusions. Self-reported voice complaints are common and functionally meaningful across diverse EDSs and hypermobility spectrum disorder populations, while direct laryngeal examination reveals a recurring substrate of CAJ instability, arytenoid prolapse, and microvascular or mucosal fragility, characteristically accompanied by preserved VF mobility. These findings are biologically coherent with the connective tissue defect that defines EDS, which may compromise voice through complementary mucosal-vibratory and articular routes, and whose clinical expression is further shaped by reflux, vocal demand, and systemic comorbidity. Voice and laryngeal involvement nonetheless remain under-recognised in routine care and underexamined in the literature. Heightened clinical awareness, multidisciplinary assessment incorporating instrumental and perceptual evaluation, and prospective standardised research are needed to define its prevalence, mechanisms, and optimal management. These conclusions should nonetheless be interpreted with caution, given that the available evidence is limited in volume and heterogeneous in design, and that no formal quality appraisal was conducted. Findings should therefore be read as a structured mapping of current knowledge.

## Figures and Tables

**Table 1:** Representative search terms organised by PCC component.

PCC	Keywords
Population	“Ehlers–Danlos syndrome”; “EDS”; “hEDS”; “Hypermobility”; “Connective tissue”
Concept	“Voice”; “Phonation”; “Dysphonia”; “Voice disorders”; “Laryngeal Dysfunction”
**Search combination**	Population terms AND Concept terms

## Data Availability

No new data were created or analysed in this study. Data sharing is not applicable to this article.

## Supplementary Materials

The following supporting information can be downloaded at: https://www.mdpi.com/article/10.3390/biology15141099/s1. Figure S1: PRISMA 2020 flow diagram for the study selection process. Table S1: Search strategy and syntax used across PubMed, Scopus, and Web of Science. Table S2: PRISMA-ScR checklist. References [51,52] are cited in the supplementary materials.

## Abbreviations

The following abbreviations are used in this manuscript:

ANS	Autonomic nervous system
CAJ	Cricoarytenoid joint
cEDS	Classical Ehlers–Danlos syndrome
clEDS	Classical-like Ehlers–Danlos syndrome
CT	Computed tomography
dEDS	Dermatosparaxis Ehlers–Danlos syndrome
EDSs	Ehlers–Danlos syndromes
EMG	Electromyography
FNE	Flexible naso-endoscopy
GERD	Gastroesophageal reflux disease
GRBAS	Grade, Roughness, Breathiness, Asthenia, Strain scale
hEDS	Hypermobile Ehlers–Danlos syndrome
HSD	Hypermobility spectrum disorder
JBI	Joanna Briggs Institute
LPR	Laryngopharyngeal reflux
MRI	Magnetic resonance imaging
MTD	Muscle Tension Dysphonia
NBI	Narrow-band imaging
OSF	Open Science Framework
PCC	Population–Concept–Context
pEDS	Periodontal Ehlers–Danlos syndrome
POTS	Postural Orthostatic Tachycardia Syndrome
PRISMA-ScR	Preferred Reporting Items for Systematic Reviews and Meta-Analyses extension for Scoping Reviews
PROMs	Patient-reported outcome measures
REDCap	Research Electronic Data Capture
SD	Standard deviation
SLP	Speech-language pathologist/pathology
TMJ	Temporomandibular joint
VF	Vocal fold
VFs	Vocal folds
vEDS	Vascular Ehlers–Danlos syndrome
VHI	Voice Handicap Index
VHI-10	Voice Handicap Index-10 (10-item version)
VHI-30	Voice Handicap Index-30 (30-item version)
