# Supplementary material for: Characterising Vocal Function and Laryngeal Structural Alterations in Ehlers–Danlos Syndromes: Insights from a Scoping Review
Source: Biology (Basel). 2026 Jul 8;15(14):1099. doi: 10.3390/biology15141099 (PMC13405989; doi:10.3390/biology15141099)
Supplement: Supplementary file 1 [file biology-15-01099-s001.zip › Table S1.pdf]

**Table S1. Search strategy and syntax used across PubMed, Scopus, and Web of Science.**

| Database | Search strategy |
|---|---|
| **PubMed** | ("Ehlers-Danlos Syndrome"[MeSH Terms] OR "Ehlers-Danlos"[tiab] OR hEDS[tiab] OR EDS[tiab] OR hypermobility[tiab] OR "hypermobility spectrum disorder"[tiab]) AND (voice[tiab] OR vocal[tiab] OR phonation[tiab] OR "voice disorders"[tiab] OR dysphonia[tiab] OR larynx[tiab] OR laryngeal[tiab] OR "laryngeal dysfunction"[tiab] OR "muscle tension dysphonia"[tiab] OR dysphagia[tiab] OR laryngospasm[tiab] OR "arytenoid subluxation"[tiab] OR "chronic cough"[tiab] OR dyspnea[tiab] OR "respiratory health"[tiab] OR "vocal performance"[tiab] OR singing[tiab] OR "voice quality"[tiab]) |
| **Scopus** | TITLE-ABS-KEY(("Ehlers-Danlos Syndrome" OR "Ehlers-Danlos" OR hEDS OR EDS OR hypermobility OR "hypermobility spectrum disorder") AND (voice OR vocal OR phonation OR "voice disorders" OR dysphonia OR larynx OR laryngeal OR "laryngeal dysfunction" OR "muscle tension dysphonia" OR dysphagia OR laryngospasm OR "arytenoid subluxation" OR "chronic cough" OR dyspnea OR "respiratory health" OR "vocal performance" OR singing OR "voice quality")) |
| **Web of Science** | TS=("Ehlers-Danlos Syndrome" OR "Ehlers-Danlos" OR hEDS OR EDS OR hypermobility OR "hypermobility spectrum disorder") AND TS=(voice OR vocal OR phonation OR "voice disorders" OR dysphonia OR larynx OR laryngeal OR "laryngeal dysfunction" OR "muscle tension dysphonia" OR dysphagia OR laryngospasm OR "arytenoid subluxation" OR "chronic cough" OR dyspnea OR "respiratory health" OR "vocal performance" OR singing OR "voice quality") |

*Note.* Filters applied: language (English); study type (humans only).
